# The wildcat *(Felis s. silvestris)* in the Mediterranean forest: sighting through photo-trapping and non-invasive hair collection for genetic purposes

**DOI:** 10.1007/s11259-024-10402-3

**Published:** 2024-05-21

**Authors:** Juan S.-E. Petisco, Patricia Sánchez-Carrasco, José Luis Fernández-García

**Affiliations:** 1 Animal Production and Food Science Department, Faculty of Veterinary Sciences, Universidad de Extremadura, Avda. Universidad S/N, 10003 Cáceres, Spain; 2 Taal en Communicatie, Radboud University Nijmegen, Nijmegen, The Netherlands

**Keywords:** Genetic validation, Non-invasive hair collection, Photo-trapping, Wildcat (*Felis silvestris silvestris*)

## Abstract

The European wildcat (*Felis silvestris silvestris*) is a mesocarnivore species widely distributed in Europe, from Eastern Europe to Portugal and from Scotland to Italy. Recent biogeographical studies of wildcat populations have endeavoured to assess in detail the various issues that pose a threat to this species, including hybridization with domestic cats. The use of non-invasive sampling methods supported by photo-trapping and some attractants has made it possible to gather genetic material for the detection of native wildcats in locally threatened populations, some of which live in the Iberian Peninsula. Testimonies of naturalists, hunters and farm workers led our team to choose specific areas in two large territories of Mediterranean forests where the presence of wildcats has been historically attested: the Almonte River basin and the Sierra de San Pedro Mountains. Between 2014 and 2018, non-invasive hair sampling was performed using valerian (V*aleriana officinalis*) as an attractant and supported by photo-trapping to guarantee the collection of genuine biological material (hair samples). The hair samples were genetically assessed by sequencing the nuclear gene IRBP (interphotoreceptor retinoid-binding protein) and the mtDNA gene ND4 (NADH dehydrogenase subunit 4). Despite the low density of wildcats, this combined protocol proved to be an applicable tool for detecting the presence of elusive wildcats and other mesocarnivore species in this remote region of southern Europe. In addition, non-invasive hair trapping contributes to the collection of genetic material from current wildcat populations. This procedure could enhance future management actions focused on collecting quality individualized biological material.

**Supplementary Information:**

The online version contains supplementary material available at 10.1007/s11259-024-10402-3.

## Introduction

Recently, a relevant part of the Southern European carnivore species has undergone a simplification process, which is derived from the local extinction of larger carnivore species, such as the wolf (*Canis lupus*) and the Iberian lynx (*Lynx pardinus*) (Prugh et al. 2009). Despite this, a different scenario has been described for the northern side of Europe (Chapron et al. 2014), with healthy and increasing populations of large predators, whose success has been supported by coordinated legislation shared by many European countries in the context of specific management practices and institutional arrangements (Chapron et al. 2014 and references therein). According to the latter authors, the most serious conservation problems can be expected in countries or regions where these large carnivores were extirpated in the past, as their return may trigger conflicting views. However, Prugh et al. (2009) described a different picture for medium-sized carnivores, suggesting that these species tend to be found more frequently in southern parts of Europe. As a result, the population status of these species is vital both as an indicator of ecosystem balance since mesocarnivores have not yet been extirpated (Crooks and Soulé 1999; Roemer et al. 2009; Rosalino et al. 2010) and as evidence of anthropogenic impact; these effects have recently been demonstrated in European wildcats by assessing their genetic structure (Nieto-Bláquez et al. 2022).

In Extremadura, the European wildcat is one of the surviving specimens of Pleistocene glaciations into the pockets of the Mediterranean peninsular refugia: the southern Iberian Peninsula, Italy and the Balkans (Mattucci et al. 2019; Von Thaden et al. 2021). Currently, this feline species is widely distributed throughout Europe, ranging from Eastern Europe to Portugal and from Scotland to Italy, except in Scandinavia (Nowell and Jackson 1996). Moreover, detailed geographical distributions were reported for the French (Say et al. 2012) and German wildcat (Nieto-Blázquez et al. 2022) populations. The wildcat qualifies as least concern (LC) in the IUCN Red List of Threatened Species (IUCN 2023). Despite this, molecular approaches have been used to detect hybrids with their domestic counterparts (Mattucci et al. 2019). The negative consequences of hybridization have been widely recognized by Rhymer and Simberloff (1996) and Gaudiano et al. (2022) as well as one of the main causes of the specie’s decline.

Invasive methods that involve the capture of wild animals (i.e. cage traps) intended for the collection of tissue and/or blood samples are sometimes prohibited (e.g. hunting law 14/2010, of December 9, in Extremadura) (see also Ripa et al. 2024), so these are increasingly being replaced by non-invasive sampling methods (Valderrama et al. 1999; Steyer et al. 2013). Even some local governments are committed to their extensive use (Ripa et al. 2024). Hair, saliva, spraint, faeces and similar remains collected from the environment (Peelle et al. 2019) are non-invasive mammal sampling tools for genetic research, most notably, for the unequivocal identification of sympatric species (Ruíz-Gonzalez et al. 2013, Buglione et al. 2020b; Ripa et al. 2024), as well as, for population research based on nuclear DNA and/or mtDNA markers (Buglione et al. 2020a; Ripa et al. 2024); for diet, prey selection or activity (Kamler et al. 2020); and for metabarcoding of the gut microbiome (Khairulmunir et al. 2023). Moreover, a sampling design based on hair trapping has been suggested to obtain suitable information at sites with low density and elusive carnivores (Say et al. 2012; Steyer et al. 2013; Devillard et al. 2014). Hair trapping has also been highlighted by a recent study based on genomic approaches for genuine wildcat detection in locally threatened populations (Mattucci et al. 2019).

Flavourings and other attractants have been used on felines to successfully collect hair samples (see Turbak 1998; Weaver et al. 2003; Thomas et al. 2005; García-Alaníz et al. 2010). Kery et al. (2011), Kilshaw and Macdonald (2011), Steyer et al. (2013) and Nussberger et al. (2014) conducted successful studies using the valerian tincture (*Valeriana officinalis*) as an attractant for hair trapping of the European wildcat (*Felis s. silvestris*) in the Blauen range of the Jura Mountains (Switzerland), Scotland, Kellerwald-Edersee National Park (Germany), and the Jura Mountains (Switzerland), respectively. The animals rub against wooden stakes sprinkled with valerian, leaving hairs attached to them. These hairs were subsequently subjected to genetic analysis. However, other studies, such as the one carried out in the Etna National Park in Sicily (Anile et al. 2009), documented no evidence of these animals leaving any biological material on the lure while also using the valerian tincture.

The present study, carried out between 2014 and 2018, utilized a non-invasive method for the collection of hair samples from wildcats that combines hair trapping with valerian tincture (*Valeriana officinalis*) as an attractant (Steyer et al. 2013; Anile et al. 2009) and improved upon this method with bags of natural valerian roots, and photo-trapping to facilitate phenotypic assessment. The goal was to assess the usefulness of these methods for obtaining hair roots from wildcats. Furthermore, we performed a genetic validation of the samples involving the sequencing of a fragment of the Interphotoreceptor Retinoid-Binding Protein (IRBP) genetic marker proposed by Oliveira et al. (2008) and of the ND4 mitochondrial gene (this study) for its later phylogenetic comparison to that of *Felis* spp. found in the Gene Bank (NCBI, USA).

## Materials and methods

### Study area

**Fig. 1:**
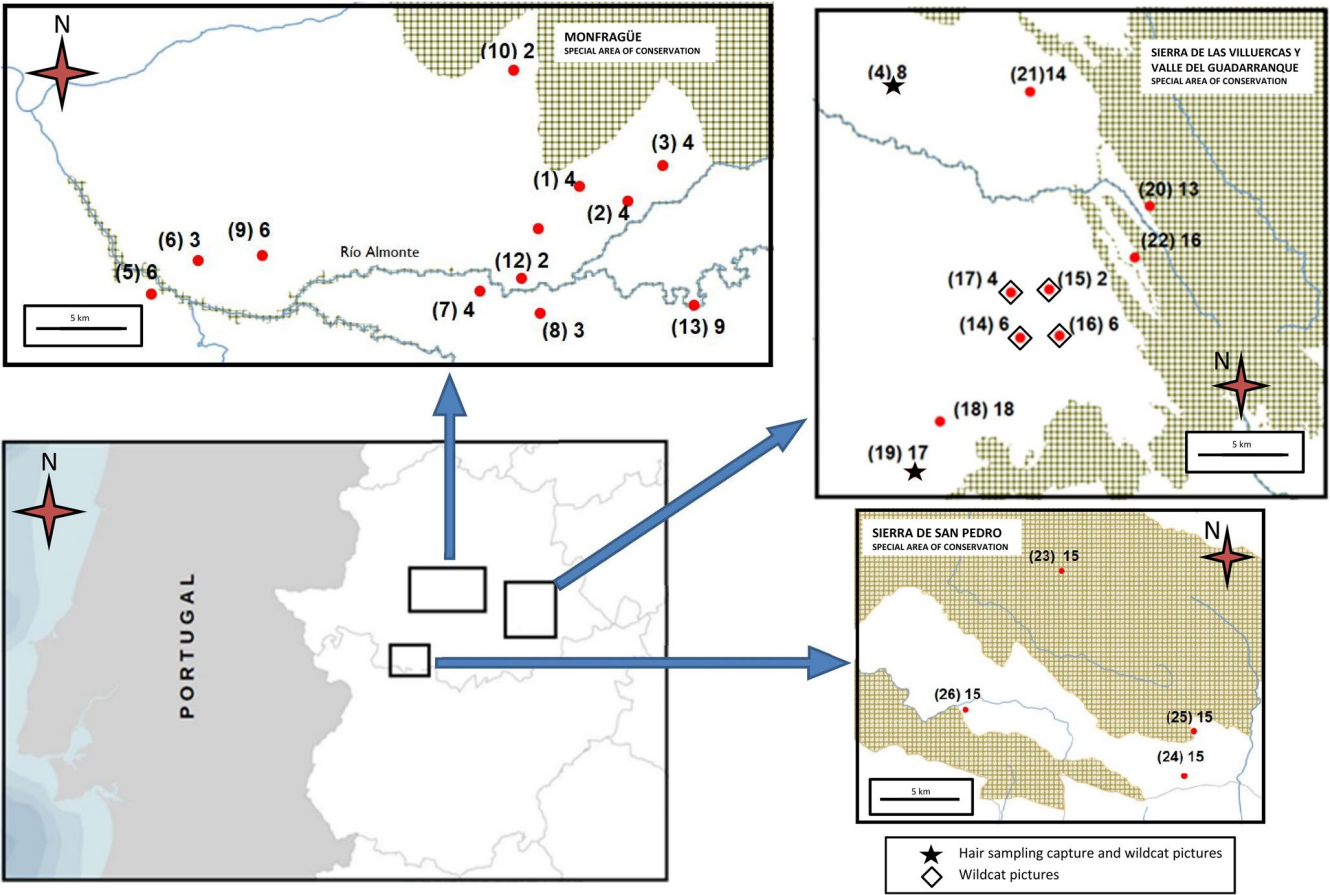
Geographical distribution of the sampling sites and results. Upper left: basin of the lower Almonte river. Upper right: higher Almonte basin river. Bottom right: Sierra de San Pedro Mountains. The red dots mark sites with no sightings of wildcats; the rhombuses mark sites with sightings of wildcats; and the stars mark sites with sightings of wildcats and wildcat hair collection.

The study was carried out in Extremadura (Spain), around the Almonte basin river (200,000 ha) and the Sierra de San Pedro Mountains (400,000 ha) areas (Fig. 1), where the presence of these felines has been confirmed at recent and historic times. This place consisted of a typical agro-sylvo-pastoral ecosystem located in the southwest quadrant of the Iberian Peninsula. This area is of great socioeconomic and conservational importance due to its exceptionally rich animal biodiversity, especially birds, and to the high value derived from grazing activities, such as the exploitation of Iberian pigs, sheep, cattle and hunting species (De Oliveira et al. 2013). As a generalization, and from an operational point of view, this area is a functional system of stockbreeding and/or hunting exploitation, with at least 50% of its pastureland surface associated with adult scattered acorn trees with variable proportions (5% to 60%) of canopy cover (Pulido and Picardo 2010).

### Photo-trapping and hair collection

During the months of December to May (i.e., mating season until the birthing season of the species) 2014–2018, fieldwork and collection of genetic material were conducted using photo trapping cameras and stakes prepared for hair collection. Stakes (dimensions: 70 cm × 5 cm × 5 cm) were placed in the field securely fixed to the ground, with their four exposed sides covered with hair-catching Velcro magic tape. At least 2/3 of the stakes were covered with hair-catching tape on each side, the top and bottom were left free, and the tape was sprayed with valerian essence (*Valeriana officinalis*) emulsified in liquid paraffin at a 1:3 ratio. Furthermore, a net bag filled with natural valerian roots was attached to the top of the stake.

The stakes were installed in forested areas with arable land where the population of prey is greater (Moleón and Gil-Sánchez 2003) as well as in shaded areas to avoid hair degradation (Piggott and Taylor 2003) and potential DNA loss due to different factors (i.e., ultraviolet rays, humid environment) (Palomares et al. 2002).

Recognition of the species was facilitated by photo-trapping (Monterroso et al. 2014; Ballesteros-Duperón et al. 2015): a TRAIL CAMERA 16 MP 1080 P 2G MMS SMS was placed 3–5 m away from each stake and programmed to shoot two photographs and a 20-s video at one-minute intervals. GPS coordinates were taken at each point (see Fig. 1). The sampling stations numbered 1 to 26 (see Table 1 and Fig. 1 for details on the sampling effort) were those for which we have historical or recent reports of sightings of wildcats. The trapping cameras worked 24 h a day for at least 15 days at a time, except for the reviews. According to the reports provided by local experts who reside, work, or visit the surveyed areas, the stations could be separated into two categories (Table 1): (1) stations where there was evidence of historical sightings of the target species from the start of sampling and (2) those stations where there was sighting in less than two consecutive reproductive periods (< 2 years) prior to the start of sampling.

**Table 1:** Photo-trapping effort by sampling year and date (month)

Sampling year	Date	Sampling stations	Photos / Videos (Nº Cameras)	Observed species	Mammals/Mesocarnivores ratio	Hair sample collection
2014/2015	December	El Tejarejo (1), Pizarro (2) y la Ventosilla (3)	91.14/45.57 (12)	1(8), 2(2), 3(4), 4(3), 7(5), 5(6),8(4), 9(9), 11(9), 12(3), 13(8), 14(4), 15(1),	52/14	
	December	El Canchal * (4)	87.25/43.63 (8)	5(1), 4(2), 7(4), 1(1), 3(3), 2(1), 8(2), 10(1), 9(5), 13(5), 15(1), 11(6), 12(4), 14(1), 16(1*)	38/8	Wildcat Leaves hairs on the stake
	January	La Perala (5) y Cabeza Gorda (6)	172.66/86.33 (9)	1(4), 3(3), 6(1), 5(1), 9(3), 14(2), 15(2), 10(5), 8(1), 11(2), 17(5), 18(2), 19(1),	32/14	
	January	Trinidade (7) y La Umbria (8)	169.14/84.57 (7)	1(6), 5(1), 18(2), 10(4), 9(2), 8(4), 17(1), 14(1), 11(2), 15(1), 12(1),	25/10	
	February	Dehesa boyal de Santiago del Campo (9)	158.33/79.17 (6)	1(4), 5(3), 6(2), 17(6), 14(1), 18(5), 10(5), 8(1), 20(1), 11(2), 9(1)	31/13	
	March	El Edén (10)	143/71.5 (2)	1(1), 15(1), 17(1), 10(1), 11(1), 9(1), 8(1),	7/3	
	April	Parapuños (11)	296.75/148.385 (8)	1(3), 3(1), 4(1), 10(6), 11(7), 18(1), 9(4), 17(2), 14(4), 12(1), 8(2), 13(1)	33/15	
	April	El Cabril (12)	50/54.5 (2)	11(1), 17(1)	2/0	
	Mai	La Gama (13)	638.88/319.4 (9)	18(1), 10(3), 11(1), 5(1)	6/4	
2015/2016	December	Valdeagudo *(14)	66/33 (6)	1(1), 5(1), 6(1), 7(1), 11(4), 8(4), 9(3), 13(3), 10(1), 18(1), **16(1)**, 15(1),	22/7	
	December	Las Mesas * (15)	44/22 (2)	16(1*) 18(1), 15(1), 10(2), 13(3), 9(3), 8(2), 11(3), 6(2), 1(1)	19/8	Wild cat Leaves hairs on the stake
	December	La Mezquita * (16)	34/17 (6)	18(1), 15(1), 17(1), 10(2), 13(3), 9(1), 8(2), 11(1), **16(1)**	13/6	
	December	El Ahijón * (17)	58/29 (4)	15(2), 14(1), 10(2), 9(2), 8(1), 11(1), 7(1), 6(1), 1(2),** 16(1)**	14/8	
	January	Moñigueros * (18)	74.22/37.11 (18)	1(4), 3(7), 9(8), 11(14), 10(11)*,* 13(8), 12(6), 15(4), 8(4)*,*	66/23	
	March	Matavacas * (19)	79.05/39.52 (17)	1(10), 2(1) 3(1), 13(11), 12(12), 11(13), 9(8), 10(4), 15(4), 8(9)*,* 18(4), 14(1), 17(2), 19(1), 16(1*)	82/24	The wild cat brushes against the down stake and leaves no hair
2016/2017	December	La Breña (20)	51.23/25.61 (13)	9(7), 10(7), 12(4), 11(5), 18(2), 17(4), 15(2), 13(2)	33/18	
	January	Valdeposadilla (21)	18.71/9.35 (14)	3(1), 4(4), 5(3), 6(2), 7(2), 13(2), 11(1), 10(5), 12(4), 9(3), 8(1), 17(1), 15(1), 14(1)	31/10	
	February	Las Paredes (22)	171.375/85.68 (16)	1(3), 13(9), 11(3), 8(3), 12(2), 10(1), 18(1), 17(1),	23/2	
2017/2018	December	La Longuera (23)	37.26/18.63 (15)	3(1), 7(2), 9(4), 10(7), 13(1), 18(2)	17/13	
	January	El Castillo de Castellano de abajo (24)	175.40/87.70 (15)	3(7), 7(3), 13(11), 10(9), 11(7), 15(5), 14(3), 9(5), *(*8(1), 18(2)	53/24	
	February	El Castillo de Castellano de arriba * (25)	204/102 (15)	3(4), 7(1), 9(11), 11(9), 10(8), 17(2), 18(5), 15(4), 14(2), 13(9), 20(1), 8(1),*)*	57/31	
	April	Barrantes (26)	60.80/30.40 (15)	*),* 2(3), 1(2), 6(1), 18(1), 11(6), 8(3), 17(2), 10(5), 13(6), 15(3), 9(7), 14(1),	40/17	The cat that appears is tabby type but does not leave hair

The sampling stations were numbered from 1 to 26 and coded depending on whether we had recent (< 2 years before the start of data collection, in bold and with asterisk) or historical (> 2 years before the start of data collection, on plain text) reports of wildcat sightings. Photos and videos averaged (number of installed cameras). Species observed by camera and period using a nomenclator (see below): number of cases in parentheses and bold numbers for wildcats (asterisk when approached the lure and/or left hair). The last two columns show the ratio of mammals *versus* mesocarnivores and the collected of wildcats hair samples, respectively. Note: Photo-trapped birds were excluded (see details Table S2)

Species observed: DOMESTIC MAMMALS: Cow (*Bos taurus*) (1); horse (*Equus caballus*) (2); sheep (*Ovis orientalis aries*) (3); goat (*Capra aegagrus hircus*) (4); dog (*Canis lupus familiaris*) (5); domestic cat (*Felis catus*) (6); pigs (*Sus scrofa domesticus*) (7). WILD MAMMALS: Hare (*Lepus europaeus*) (8); fox (*Vulpes vulpes*) (9); beech marten (*Martes foina*) (10); wild boar (*Sus scrofa ferus*) (11); roe deer (*Capreolus capreolus*) (12); red deer (*Cervus elaphus*) (13); European mongoose (*Herpestes ichneumon*) (14); badger (*Meles meles*) (15); wildcat (*Felis s. silvestris*) (16); mouse (*Mus musculus*) (17); genet (*Genetta genetta*) (18); rabbit (*Oryctogalus cuniculus*) (19); and otter (*Lutra lutra*) (20)

During maintenance and review of the study stations, in the stakes where evidence of rubbing was found, forceps and magnifying glass were used to collect the material (hair samples). The samples were inspected to exclude hairs not stemming from carnivores, as evidenced by their morphology, using an identification key (Teerink 1991) and preserved in labelled plastic zipper bags with dried, UV-sterilized silica micro bags. The photo-trapping sites were moved to a new position or station every 30–45 days, considering the information provided by the sightings of the species. The cameras (24 h with 8–16 GB memory cards) were checked, and the stakes were controlled, including the collection of biological material. This process was carried out every seven to fifteen days (shorter intervals on rainy days).

We conducted a Wilcoxon signed-rank test to determine whether significant differences existed in the patterns of distribution of mesocarnivores in areas where wildcats were sighted recently. We contrasted the total number of mesocarnivores photographed at all stations with the total number of mesocarnivores photographed only at stations with reported sightings of wildcats in the last two years. The test was performed using SPSS for Windows version 15.0 (Chicago, SPSS, Inc., under the UNEX licence).

### DNA extraction, primers selection, amplification, and sequencing

DNA were obtained of the hair samples (4–5 bulbs) collected from both wildcat (*Felis s. silvestris*). DNA from one domestic cat (Mainecoon breed) and one dog (*Canis lupus familiaris*) was used as PCR positive controls. The DNA was extracted using the commercial G-spin™ Total Kit (INtRON Biotechnology) following manufacturer’s instructions (protocol H for hair). The DNA from the dog was included because reference sequence from *Canis familiaris* was used in the primer design of the nuclear IRBP gene. As in Oliveira et al. (2008), the partial nuclear gene IRBP used expand nucleotide positions from 35288297 to 35288057 of the sequence with Acc. Number HG994386.1 of the *Canis lupus* genome (chromosome 4). This portion contains the first exon of the IRBP3 CDS (coding sequence). Primer3plus software (Untergasser et al. 2012) was used to design the following primers: IRBPjl-F 5ʹ-GGCCAGTCYGAYTTCTTCCT-3ʹ and IRBPjlR 5ʹ-GCCCGYACCAGGAGCC-3ʹ. Nucleotide positions with ambiguities were introduced to amplify this segment in all mesocarnivores of the Iberian Peninsula, as recommended by Oliveira et al. (2008). These primers were used to amplify and sequence a partial fragment of the IRBP nuclear gene with 354 bp.

Furthermore, a mitochondrial NADH dehydrogenase subunit 4 gene was amplified with the primers FSIND4-F 5ʹ-CTAGCCAGCATARCCCCCATT-3ʹ and FSIND4-R 5ʹ-GGTTGAGATRGGCTTGGCT-3ʹ These primers were designed (Primer3plus software, Untergasser et al. 2012) and selected based on an in silico comparison of the complete mitochondrial genome of *F. s. silvestris*, including *F. s. bieti* and *F.s. lybica* (Acc. N^o^ KP202278, KP202273, KP202275, respectively) and *F. catus* (Acc. N° MT499915 and NC_001700). The sequences comprised a fragment of 692 bp.

All PCRs were performed in a 15 µl reaction mixture containing 1.5 µl of genomic DNA (~ 2–5 ng), 1.5 µl of 10 µM of each primer, 10X buffer (1.5 mM Cl2Mg), 2 mM DNPs, and 0.3 units of BIOTAQ™ polymerase (Meridian Life Science) in separated reactions of IRBP and ND4, respectively. The amplification reactions were performed in a 2720 Thermal Cycler (Thermal Cycler from Applied Biosystems®) following one initial cycle of 5 min at 95 °C; 38 or 32 cycles of 0.5 min at 95 °C, 1 min at 55 ºC or 52 °C (melting temperature for the IRBP and ND4 gene, respectively) and 1 min at 72 °C; and a final cycle with an extension incubation of 10 min at 72 °C (the number of cycles was 38 or 32 for the IRBP and ND4 gene, respectively). PCR products were checked after electrophoresis on a 1.5% agarose gel stained with SYBR® safe dye (Invitrogen, Thermo Fisher Scientific) in a UV transilluminator. Amplified products (10 µL) were purified by ExoSap-IT (Thermo Scientific) according to the manufacturer’s recommendations. Big Dye® 3.1 cycle sequencing kit (Thermo Fisher Scientific) was used to sequence the products following Applied Biosystems recombination in both directions. DTR gel filtration cartridges (Edge Biosystems, Gaithersburg, MD) were used to remove residual dye molecules that cause the presence of dye-blobs. Sequence profiles were obtained in a genetic DNA analyser (Applied Biosystems™ 3130 DNA Analyser) using the computer application “ABI Sequencing Analysis” version 5.2 (Applied Biosystems Company, USA).

### Sequence data analysis

Regarding the nuclear IRBP gene, because three heterozygous sites (polymorphic sites) were found in some of the sequences, those sites were manually coded using IUPAC codes and aligned in MEGA 6.06 software (Tamura et al. 2013). Seven sequences from *Felis* spp. found by “BLAST search” at NCBI, were added to the three IRBP sequence of this study. These seven sequences were as follows: *F. s. silvestris* (*n* = 2, Acc. N° AY170072.1 and GQ214060.1), *F. s. bieti* (*n* = 1, Acc. N° AY525033.1) and *F. catus* (*n* = 4, Acc. N°: AP023162.1, XM_003994154.4, GQ214061.1 and XM_019813453.2). After removing primers from the sequences, all the sequences (*n* = 10) were aligned to identify both intra- (heterozygous individuals) and inter-sequence variations. These sequences (genotypes) were used for the reconstruction of the minimum number of haplotypes in the data set with the "open unphase/genotipe data file” option in DnaSP version 6.12.03 (Rozas et al. 2017). After, the haplotype diversity (h), nucleotide diversity per site (π) and recombination events were obtained in this software.

With respect to the mitochondrial gene, after filtering the NCBI database for the genus *Felis,* 120 sequences of the ND4 segment (NADH dehydrogenase subunit 4) were found. All but one of these sequences were downloaded because they exhibited 100% sequence coverage (618 bp in size) with respect to those in this study. Finally, a total of 122 sequences remained. These sequences were collapsed into haplotypes using DnaSP. These haplotypes were further used both to determine the number of fixed polymorphic site differences and to create a standard Median-Joining (MJ) Network (Bandelt et al. 1999) to visualize the genetic relationships between sequences with the program PopArt v. 1.7 (Population Analysis with Reticulate Trees) (Leigh and Bryant 2015). The MJ haplotype network was pictured considering the following traits: FSS, BIETI, FSLCAT, CAT, MARGARIT, NIGRIP and CHAUS for *F. s. silvestris*, *F. s. bieti*, *F.s. lybica*, *F. catus, F. margarita*, *F. nigripes* and *F. chaus*, according to the taxonomic assignments of the reference sequences (see supplementary material, Table S1).

## Results

### Hair collection

The information obtained from the trapping cameras, after excluding birds and cattle also attracted by the lure, included 270 images and 135 videos of mesocarnivore species (Table 2), which were recorded at a total of 26 sampling stations (fences). There were 143 images for stations with sightings in the last 2 years (marked in bold and with asterisk in Table 1).

**Table 2:** Mesocarnivores (MC) species detected by photo-trapping as follows: photo-trapped MC species

SPECIES	Total < 2 years (MC)	Total MC	Ratio < 2 years (A)	Total ratio (B)	Ratio = (A/B)–- 1
FOX 22	41	87	0.357	0.321	0.11
BEECH MARTEN 23	31	89	0.270	0.328	-0.18
EUROPEAN MONGOOSE 27	5	22	0.043	0.081	-0.46
BADGER 28	18	34	0.157	0.125	0.25
WILDCAT 29	6	6	0.052	0.022	1.36
GENET 31	13	31	0.113	0.114	-0.01
OTTER 33	1	2	0.009	0.007	0.18
TOTAL	115	271	1.000	1.000	

Total number of MC photo trapped in sites with recent sightings (< 2 years) of wildcats. Total number of MC sampled at all stations. Ratio of each MC species photo-trapped at sites with recent sightings (< 2 years) to the total number of MC species (A). Ratio of each MC species contained in all stations to the total number of MC species (B). Ratio (A/B)-1

Six different stations showed wildcats images (sampling stations: 4, 14, 15, 16, 17 and 19; Table 1). As a result, wildcats were photo-trapped in 23.1% of the total number of stations surveyed. However, the species was found at 75% (6/8) of the stations with sightings in the last 2 years and uniquely in the Almonte basin area. The coat and morphological patterns observed in the pictures suggested that the taxonomic identification was compatible with the wildcat (*Felis s. silvestris*) (Ragni and Possenti 1996). The overall success rate in the capture of photos (Table 2), excluding domestic cats, was 2.2% for the general category (without distinguishing by history of the sampled stations), but it doubled (4.2%) when mesocarnivores pictures were considered at only the eight stations with recent sightings (Table 1).

**Fig. 2:**
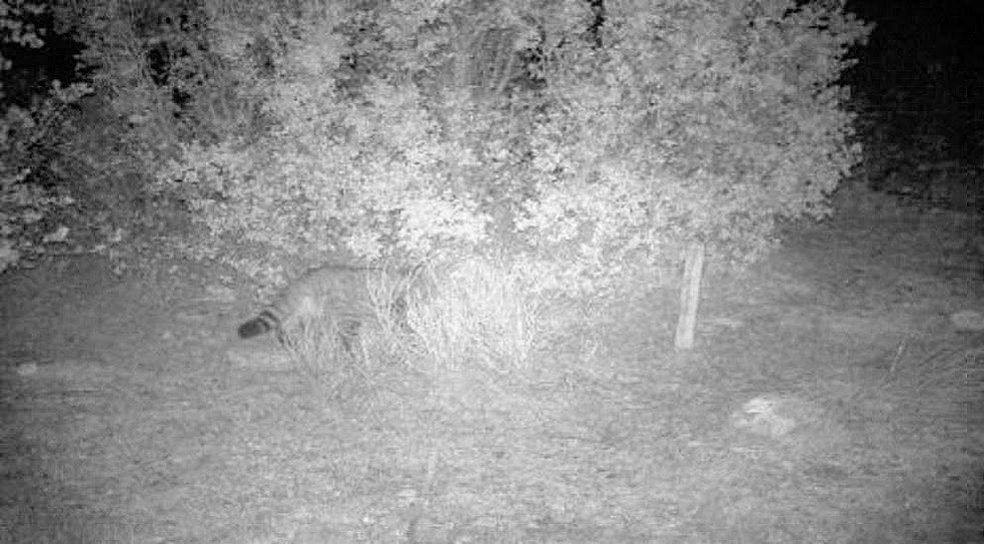
Wildcat *(Felis s. silvestris)* surrounding Deleitosa. This wildcat left hair on the stake

A total of 33.3% (2/6) of the wildcats captured by the trapping cameras left hair samples on the stakes (Table 1). Unfortunately, a third cat rubbed against the stake, but it was torn down in a storm, and the genetic material could not be collected properly (Fig. 2).

In addition to the wildcat data, hair collection from other mesocarnivore species was reported from the Extremadura Forest (data not shown). The division of the fences into recent (less than 2 years since the start of the study) and historical (over 2 years since the start of the study) (Tables 1 and 2) showed the relevance of collecting this information to increase the number of photo-trapped wildcats. A significant Wilcoxon range test (Z = -2.201; sig Monte Carlo bilateral *p* value = 0.035) suggested possible differences in the distribution of mesocarnivores when wildcats, even at low density, were present (details in Materials and methods and Figure S1).

**Fig. 3:**
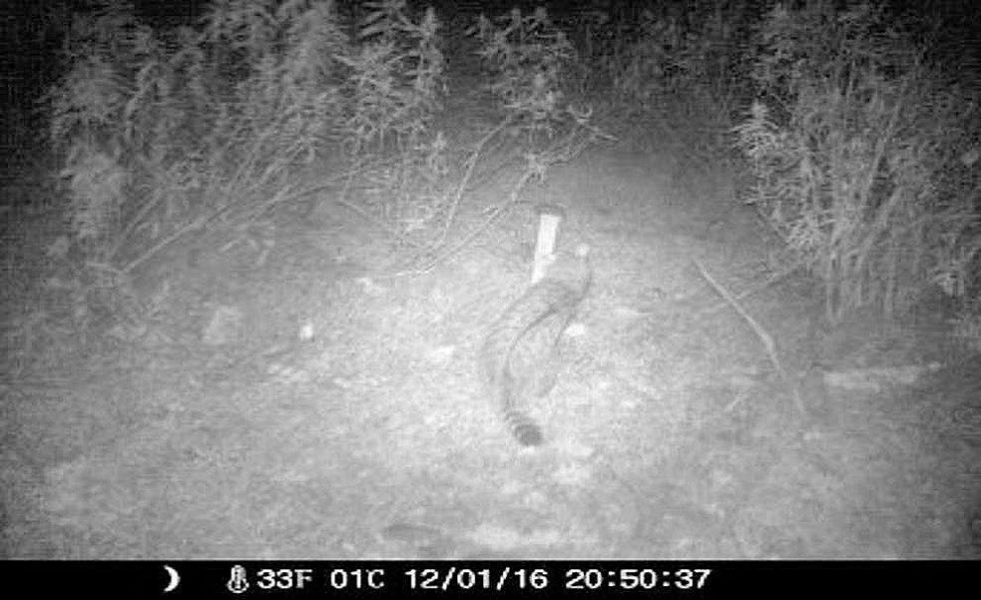
Wildcat (*Felis s. silvestris*) surrounding the Garciaz river. Leaf hair

**Fig. 4:**
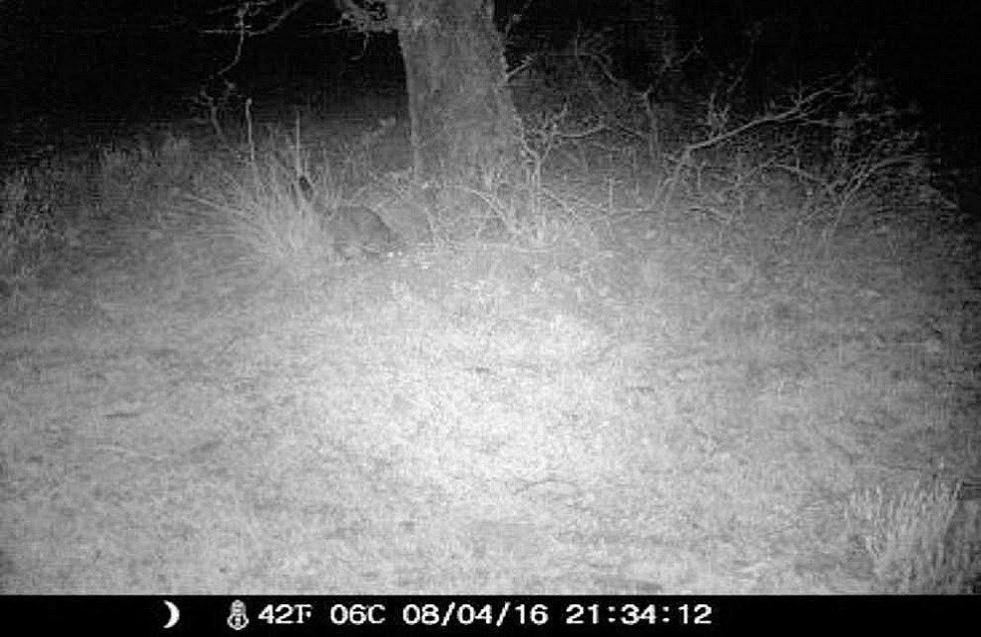
Wildcat *(Felis s. silvestris)* surrounding the Garciaz river. Leaves no hair

Our non-invasive design resulted in a nonnegligible proportion of photo-trapped wildcats (2/6) leaving enough hairs (at least 4–5 hairs per collection) (Figs. 3 and 4) to obtain genetic material. Moreover, although anecdotal, a photo-trapped female, possibly pregnant due to her obvious gestation state (Figure S4), was filmed 200 m from the photo-trapping post with her cubs a few months later (Figure S5 and S6; Video-trapping S3).

### Genotyping and validation

To identify the species to which the hair belonged, the two samples were genetically studied by sequencing both the IRBP gene and a partial portion of the overlapping mitochondrial ND4L and ND4 gene. The sequences were deposited in the Gene Bank (Acc. Nº OQ096703, OQ096704, OQ096705 and OQ096700; OQ096701; and OQ096702, with respect to IRBP and ND4 sequences, respectively), belonging to the Mainecoon domestic cat and the two samples collected from wildcats. Analysis of the IRBP gene revealed a total of 3 SNP sites (Table 3), one of which was synonymous (at 354 aa: wildtype/non wild type: G/A) and two of which were nonsynonymous at D355A (wild type/non wild type: A/C) and L363V (wild type/non wild type: G/C), which contained amino acid residue changes. For the first time, after reconstructing the haplotype from IRBP genotype data (three from this study and seven from GenBank) five haplotypes were obtained (Hap 1 to Hap 5; Table 3), Hap 1 was the only one observed in wildcat (*F. s. silvestris* and *F. s. bieti*) specimens. However, all other haplotypes were only found in the domestic cat genotypes. According to DnaSP results, two recombination events were detected using the IRBP sequences (Table 3). All four possible gametes were present (details for haplotypes and gametes are shown in Table 3). The diversity of these datasets was studied. The haplotype diversity was h = 0.558 (*SD* = 0.114), and the per-site nucleotide diversity was π = 0.004 (*SD* = 0.001). The h and π values decreased to zero for wildcats after all declared domestic cats were removed. The values (h and π) increased to h = 0.800 (*SD* = 0.100) and π = 0.005 (*SD* = 0.005) when *F. s. silvestris* was excluded, suggesting greater diversity in the domestic counterpart.

**Table 3:** IRBP haplotypes, sequence (SNP variation), gamete recombination event, frequency, and species after reconstruction via DnaSP software

	Sequence	Gamete event 1	Gamete even 2	Frequency	Specimen
Hap1	GAG	GA	AG	13	Wild * and domestic cat ^#^
Hap2	AAC	AA	AC	1	domestic cat^#^
Hap 3	ACC	AC	CC	4	domestic cat^#^
Hap 4	GCG	GC	CG	1	domestic cat^#^
Hap 5	GCC			1	domestic cat^#^

* or # for wild cats and domestic cats from this study, respectively

These 122 ND4 sequences collapsed into 20 haplotypes. These 20 haplotypes were grouped to determine DNA divergence in polymorphic sites between pairs of taxon groups (for groups details, see Table 4). One hundred and four polymorphic (segregating) sites were observed among the twenty ND4 haplotypes, five of which exhibited fixed differences between domestic and other wild cats (*Felis catus* + *F.s. lybica* and *F.s. bieti*) regarding European wildcats (*F.s. silvestris*). This difference decreased to two sites when seventeen of the haplotypes were compared to the three wildcat haplotypes (Table 4). Interestingly, two nonsynonymous changes were found in the T89M and M170T amino acid sequences (this study/OR095103: T/C and reference sequence KP202278/this study: T/C) of the ND4 sequences of *F. s. silvestris*, suggesting that further research is still worthwhile. Thus, the sequence obtained from both samples matched a maternal ancestor of the European wildcat.

**Table 4:** Polymorphic site differences between sequences of the 20 mtDNA haplotypes

	Mutations			
Polymorphic sites between sequence set (Total polymorphic sites = 104)	Nfd	A	B	C
(a) *F. s. silvestris vs* (b) *Felis catus*	6	2	22	0
(a) *F. s. silvestris vs* (c) *Felis catus* + *F.s. lybica and bieti*	5	2	23	0
(a) *F. s. silvestris vs (*d) *Felis spp no F. s. silvestris*	2	2	101	0

DNA divergence between taxon groups in columns as follows: Nfd, A, B and C columns for number fixed polymorphic differences, mutations polymorphic in (a) but monomorphic in (b, c or d), mutations polymorphic in (b, c or d), but monomorphic in (a), and shared mutations, respectively

Number of haplotypes (n) within groups: (a) *F. s. silvestris* (*n* = 3); (b) *Felis catus* (*n* = 12); (c) *Felis catus* + *F. s. lybica* and *F. s bieti* (*n* = 13); (d) *Felis* spp but no *F. s. silvestris* (*n* = 17)

**Fig. 5:**
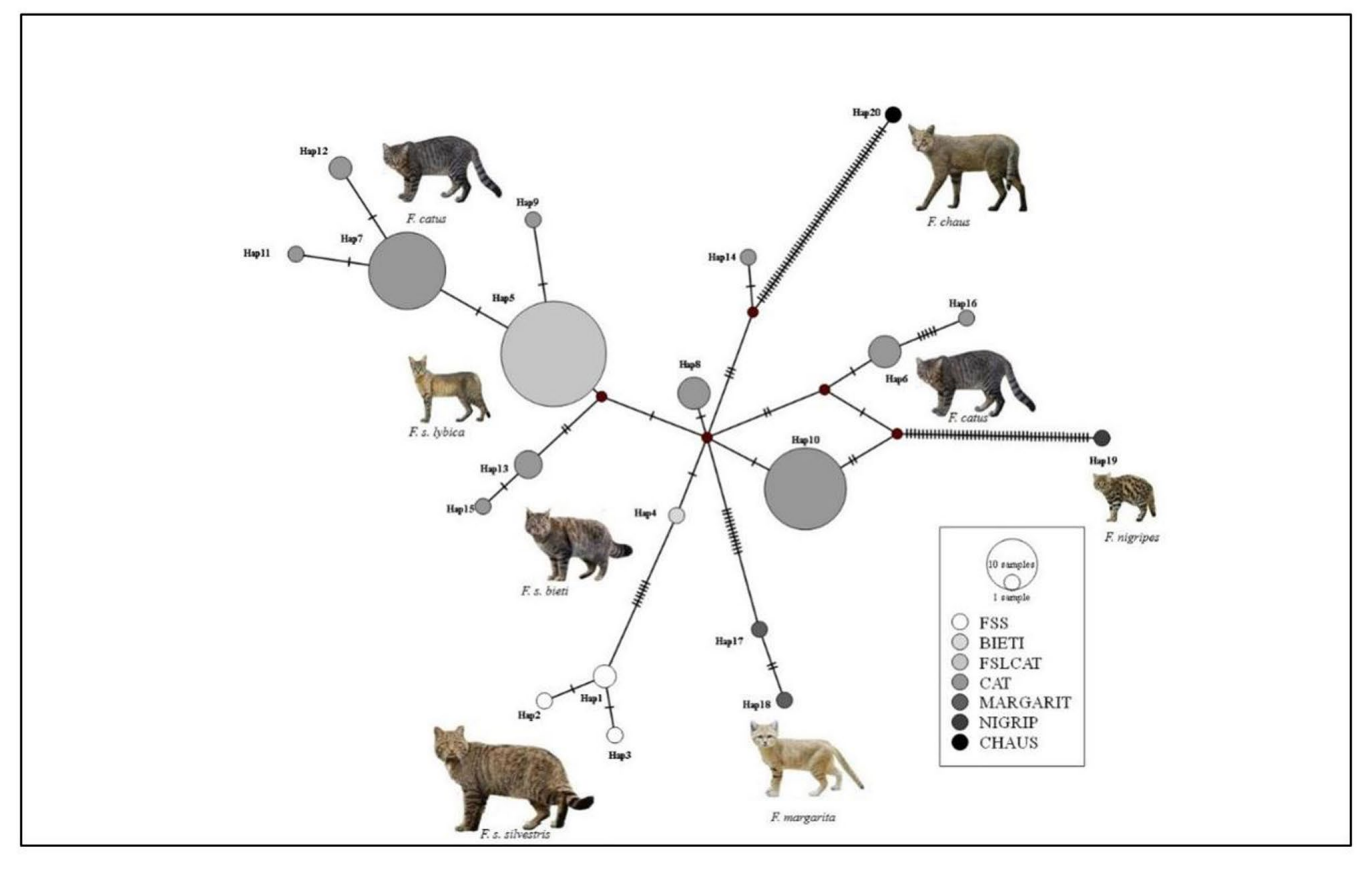
MJ networks among twenty ND4 haplotypes (details in supplementary material Table S1). The node size (haplotype) was proportional to the frequency of the sequences. The color plot was pictured following the trait groups (details in the Materials and methods section). The small bars indicate the number of mutations (greater than one) between two different haplotypes

Figure 5 shows the haplotype network, which included three main clades for domestic cats (hap5, hap7 and hap10), similar to those observed in previous studies (Patterson et al. 2023 and reference therein) and despite the number of ND4 informative sequences was reduced by approximately one-fifth with respect to the complete mitogenome. Furthermore, haplotype 5 was shared between *F. s. lybica* and domestic cats (called FSLCAT; Fig. 5), suggesting more gene flow of mitochondrial lineages between African wild cats and domestic cats than with European wild cat lineages, yet. One notable feature emerged from the haplotypes of *F. s. Silvestris* for two reasons. On the one hand, the absence of mtDNA sequences shared with domestic cats, including *F. s. lybica* wildcats, was clearly demonstrated. On the other hand, the three European wild cat haplotypes differed from each other by a pair of bases which were responsible for non-synonymous changes.

## Discussion

### Photo-trapping for non-invasive sampling

Our results demonstrated the low density of the species in the area, in line with Gómez-Chicano et al. (2020) and in support of the need for greater efforts to implement photo-trapping to locate them (Steyer et al. 2013). Despite this low-density conditions, biological material collection was more successful after conducting a survey prior to choosing the area of study, as suggested for scarce, elusive, or cryptic species (Fernández-García and Vivas-Cedillo 2017).

Despite having used shorter camera exposure times than Anile et al. (2012a, b) did at each of the sampling stations, we succeeded in choosing the attractant and timing. Anile et al. (2010) explained the absence of results in their study because they carried out their fieldwork outside of the mating season (during November/December), suggesting that the mating season is relevant under reduced censuses. As supported by Kery et al. (2011), longer periods of photo-trapping, especially between December and May, improved both hair sample collection and photo-trapping capture at low density (Steyer et al. 2013; Gil-Sánchez et al. 2020). However, studies conducted in Scotland (Kilshaw and Macdonald 2011) and Sicily (Anile et al. 2012a) did not obtained positive results because no differences existed using different scenarios: day or night, setting the lure or not, having cameras with or without flash, or the presence or absence of the attractant over a long or short period of time (Anile et al. 2009, 2010, 2012a).

Furthermore, a remarkable finding of this study was the action of the attractant on different species, including domestic cats (e.g., Figure S15), domestic livestock and wild mammals, which also left hair (see Figures S7 to S15) for further genetic tests. In addition, photo-trapping has proven to be a method that minimizes disturbances to an animal or its natural behaviour and increases the probability of detecting wildcats (Jerosch et al. 2017; Monterroso et al. 2014).

### Genotypic assessment

Several molecular methodologies have been developed to evaluate species discrimination between domestic cats and European wildcats. Polymorphic microsatellites and diagnostic SNPs have recently been considered complementary molecular approaches (Nussberger et al. 2023) for assessing hybridization between domestic and wild cats (Nussberger et al. 2014; Mattucci et al. 2019). In this study, three SNP sites of the IRBP *locus* showed genetic variation between domestic cats and wildcats, but it does not clearly distinguish between the two species. However, the wild-type haplotype was the only one found in *F. s. silvestris* (Table 3). Oliveira et al. (2008) also found intraspecific variation in this nuclear gene in this species (Acc. Nº GQ214060 to GQ214061), which does not contradict our findings. Although hybridization was not our objective and there is not enough data, we would like to highlight the relevance of the nuclear haplotypes consisting of blocks of SNPs such as those found in this study. The three polymorphic positions of the IRBP gene were tightly linked, spanned a maximum of 16 nucleotides and could therefore be considered a bin or linkage block in the mapping jargon. These finding merits support from a well-detailed linkage study with adequate sampling, as extensively reviewed by Sved and Hill (2018). Because IRBP haplotypes showed all possible gametes, additional reports on this gene could help uncover whether recombination occurs, which may serve to assess LD in admixed populations (Sved and Hill 2018). According to the data collected in this study, only five independent allele blocks (haplotypes) might be supported at this *locus* (Table 3). However, since wild and domestic cats share only one IRBP haplotype, all the other haplotypes should not be found within the populations of European wildcat populations to be reasonably excluded from them. But it will be necessary to demonstrate it through a broader and more exhaustive analysis.

Furthermore, mitochondrial information has also been considered to be of complementary interest in the support of hybridization issues due to its mode of inheritance (Nussberger et al. 2023). With respect to the SNP chip used to detect accurate genotyping of wildcat introgression from domestic cats, Nussberger et al. (2014) proposed seven polymorphism sites in the ND5 (NADH dehydrogenase subunit 5) mitochondrial gene (mtDNA SNPID1 to mtDNA_SNPID4 and mtDNA_SNPn to mtDNA_SNPx). However, one of these seven SNPs (SNPx) showed a 100% match to a numt located at chromosome D2 (Acc. Number AP023162) in the genome of *Felis catus* (Senzu breed). No numt was detected in the ND4 gene (Patterson et al 2023, this study), suggesting that at least 2 fixed SNPs among the analysed *Felis* (Table 4) species may be useful for wildcat assessments in a new SNP chips. As suggested in Velli et al. (2023), the use of a highly diagnostic mtDNA segment may be justified when admixture patterns in European wildcats are suspected to be female biased. Highly diagnostic mtDNA sequences have been used to support the matrilineal exchange of mitochondria between domestic cats and European wildcat populations (Patterson et al. 2023), assuming that their behaviour is a selectively neutral polymorphism. Thus, mtDNA variation provides a tool for an exhaustive description of the phylogenetic and phylogeographic structure of European wildcats across the entire species range (Velli et al. 2023; Patterson et al. 2023). However, it is pertinent to report an unforeseen outcome of nonsynonymous intraspecific variation among the three wildcat sequences for the ND4 gene, which may also be suggestive to ask whether these changes could be relevant to the function of mitochondrial genes.

## Conclusion and management implications

This study demonstrates that less cruel methods help to collect valuable biological material for genetic studies of wildcats (*Felis s. silvestris*), supporting its outstanding usefulness as a sampling method in management actions even at low density of this species. In addition, photo-trapping is a highly recommended way of simultaneously obtaining genetic material and images of the phenotype of the individual concerned. In fact, it has also been recognised as a non-invasive sampling method for other populations of mesocarnivore species (Steyer et al. 2013; Mattucci et al. 2019), after adapting it to the particularities of such species.

### Supplementary Information

Below is the link to the electronic supplementary material.

Supplementary file1 (DOC 78 kb)

Supplementary file2 (DOCX 18 kb)

Supplementary file3 (DOCX 271 kb)

Supplementary file4 (DOCX 344 kb)

## Data Availability

Sequence data that support the findings of this study have been deposited in the NCBI data base (acc N are  inside the articul).
