# Supplementary material for: The wildcat (Felis s. silvestris) in the Mediterranean forest: sighting through photo-trapping and non-invasive hair collection for genetic purposes
Source: Vet Res Commun. 2024 May 21;48(4):2309–20. doi: 10.1007/s11259-024-10402-3 (PMC11315778; doi:10.1007/s11259-024-10402-3)
Supplement: Supplementary file 1 — Supplementary file1 (DOC 78 kb) [file 11259_2024_10402_MOESM1_ESM.doc]

**Table S1: Haplotypes frequency, grouping for POPART and its corresponding acc. Number and author reports.**

**Haplotypes found.**

[Hap# Freq. Sequences]

[Hap_1: 2 1-2]

[Hap_2: 1 3]

[Hap_3: 1 4]

[Hap_4: 1 5]

[Hap_5: 43 6-48]

[Hap_6: 4 49 53 94 101]

[Hap_7: 23 50 52 57-58 60-61 63 65 67-70 72-73 80 82 87 91 93 95-98]

[Hap_8: 4 51 76 85 104]

[Hap_9: 1 54]

[Hap_10: 26 55-56 59 62 64 66 71 74-75 77-79 81 83-84 86 88-90 92 99-100 102-103 105-106]

[Hap_11: 1 107]

[Hap_12: 2 108-109]

[Hap_13: 3 110-112]

[Hap_14: 1 113]

[Hap_15: 1 114]

[Hap_16: 1 115]

[Hap_17: 1 116]

[Hap_18: 1 117]

[Hap_19: 1 118]

[Hap_20: 1 119]

**POPART Groups for Felis genus, including authors reports in NCBI (in bold from this study)**

1. FSS (*Felis silvestris silvestris*)

[Hap_1: 2 **OQ096701.1:1-618_Felis_silvestris_silvestris_isolate_FSS2-Las_Mesas_NADH_dehydrogenase_subunit_4_(ND4)_gene_partial_cds_mitochondrial**

**OQ096700.1:1-618_Felis_silvestris_silvestris_isolate_FSS1-Canchal_NADH_dehydrogenase_subunit_4_(ND4)_gene_partial_cds_mitochondrial**]

[Hap_2: 1 OR095103.1:10694-11311_Felis_silvestris_mitochondrion_complete_genome]

[Hap_3: 1 NC_028310.1:10780-11397_Felis_silvestris_isolate_FSI_mitochondrion_complete_genome]

1. BIETI (*Felis silvestris bieti*)

[Hap_4: 1 KP202273.1:10949-11566_Felis_silvestris_bieti_isolate_FBI_mitochondrion_complete_genome]

1. FSLCAT (*Felis silvestris lybica* and domestic cats sharing this haplotype)

[Hap_5: 43 OR085763.1:10690-11307_Felis_catus_isolate_Cat_P1_mitochondrion_complete_genome

OR085762.1:10690-11307_Felis_catus_isolate_Cat_P19_mitochondrion_complete_genome

OR085761.1:10690-11307_Felis_catus_isolate_Cat_P11_mitochondrion_complete_genome

OR085760.1:10690-11307_Felis_catus_isolate_Cat_G_mitochondrion_complete_genome

OR085758.1:10690-11307_Felis_catus_isolate_Cat_97_mitochondrion_complete_genome

OR085755.1:10690-11307_Felis_catus_isolate_Cat_70_mitochondrion_complete_genome

OR085754.1:10690-11307_Felis_catus_isolate_Cat_65_mitochondrion_complete_genome

OR085746.1:10690-11307_Felis_catus_isolate_Cat_15_mitochondrion_complete_genome

OR085740.1:10690-11307_Felis_catus_isolate_Cat_110_mitochondrion_complete_genome

OR077857.1:10690-11307_Felis_catus_isolate_Cat_P8_mitochondrion_complete_genome

OR077854.1:10690-11307_Felis_catus_isolate_Cat_P5_mitochondrion_complete_genome

OR077853.1:10690-11307_Felis_catus_isolate_Cat_P17_mitochondrion_complete_genome

OR077852.1:10690-11307_Felis_catus_isolate_Cat_P16_mitochondrion_complete_genome

OR077851.1:10690-11307_Felis_catus_isolate_Cat_P14_mitochondrion_complete_genome

OR077849.1:10690-11307_Felis_catus_isolate_Cat_BCB34_mitochondrion_complete_genome

OR077839.1:10690-11307_Felis_catus_isolate_Cat_90_mitochondrion_complete_genome

OR077834.1:10690-11307_Felis_catus_isolate_Cat_83_mitochondrion_complete_genome

OR077830.1:10690-11307_Felis_catus_isolate_Cat_79_mitochondrion_complete_genome

OR077829.1:10690-11307_Felis_catus_isolate_Cat_78_mitochondrion_complete_genome

OR077827.1:10690-11307_Felis_catus_isolate_Cat_76_mitochondrion_complete_genome

OR077820.1:10690-11307_Felis_catus_isolate_Cat_68_mitochondrion_complete_genome

OR077818.1:10690-11307_Felis_catus_isolate_Cat_64_mitochondrion_complete_genome

OR077815.1:10690-11307_Felis_catus_isolate_Cat_60_mitochondrion_complete_genome

OR077812.1:10690-11307_Felis_catus_isolate_Cat_56_mitochondrion_complete_genome

OR077809.1:10690-11307_Felis_catus_isolate_Cat_50_mitochondrion_complete_genome

OR077807.1:10690-11307_Felis_catus_isolate_Cat_48_mitochondrion_complete_genome

OR077806.1:10690-11307_Felis_catus_isolate_Cat_47_mitochondrion_complete_genome

OR077804.1:10690-11307_Felis_catus_isolate_Cat_45_mitochondrion_complete_genome

OR077802.1:10690-11307_Felis_catus_isolate_Cat_43_mitochondrion_complete_genome

OR077801.1:10690-11307_Felis_catus_isolate_Cat_42_mitochondrion_complete_genome

OR077800.1:10690-11307_Felis_catus_isolate_Cat_41_mitochondrion_complete_genome

OR077798.1:10690-11307_Felis_catus_isolate_Cat_39_mitochondrion_complete_genome

OR077797.1:10690-11307_Felis_catus_isolate_Cat_38_mitochondrion_complete_genome

OR077796.1:10690-11307_Felis_catus_isolate_Cat_37_mitochondrion_complete_genome

OR077791.1:10690-11307_Felis_catus_isolate_Cat_30_mitochondrion_complete_genome

OR077790.1:10690-11307_Felis_catus_isolate_Cat_2_mitochondrion_complete_genome

OR077780.1:10690-11307_Felis_catus_isolate_Cat_17_mitochondrion_complete_genome

OR077778.1:10690-11307_Felis_catus_isolate_Cat_13_mitochondrion_complete_genome

OR077772.1:10690-11307_Felis_catus_isolate_Cat_112_mitochondrion_complete_genome

OR077771.1:10690-11307_Felis_catus_isolate_Cat_111_mitochondrion_complete_genome

OR077770.1:10690-11307_Felis_catus_isolate_Cat_108_mitochondrion_complete_genome

OR077768.1:10690-11307_Felis_catus_isolate_Cat_106_mitochondrion_complete_genome

KP202275.1:10875-11492_Felis_silvestris_lybica_isolate_FLI_mitochondrion_complete_genome]

1. CAT (*Felis catus*)

[Hap_6: 4 OR085764.1:10984-11601_Felis_catus_isolate_Cat_67_mitochondrion_partial_genome OR085741.1:10690-11307_Felis_catus_isolate_Cat_113_mitochondrion_complete_genome

OR077786.1:10690-11307_Felis_catus_isolate_Cat_26_mitochondrion_complete_genome

OR077775.1:10690-11307_Felis_catus_isolate_Cat_118_mitochondrion_complete_genome]

[Hap_7: 23 OR085753.1:10690-11307_Felis_catus_isolate_Cat_62_mitochondrion_complete_genome OR085745.1:10690-11307_Felis_catus_isolate_Cat_124_mitochondrion_complete_genome

OR077848.1:10690-11307_Felis_catus_isolate_Cat_BCB33_mitochondrion_complete_genome

OR077847.1:10690-11307_Felis_catus_isolate_Cat_BCB32_mitochondrion_complete_genome

OR077845.1:10690-11307_Felis_catus_isolate_Cat_A_mitochondrion_complete_genome

OR077844.1:10690-11307_Felis_catus_isolate_Cat_9_mitochondrion_complete_genome

OR077842.1:10690-11307_Felis_catus_isolate_Cat_95_mitochondrion_complete_genome

OR077837.1:10690-11307_Felis_catus_isolate_Cat_88_mitochondrion_complete_genome

OR077835.1:10690-11307_Felis_catus_isolate_Cat_85_mitochondrion_complete_genome

OR077832.1:10690-11307_Felis_catus_isolate_Cat_80_mitochondrion_complete_genome

OR077831.1:10690-11307_Felis_catus_isolate_Cat_7_mitochondrion_complete_genome

OR077828.1:10690-11307_Felis_catus_isolate_Cat_77_mitochondrion_complete_genome

OR077825.1:10690-11307_Felis_catus_isolate_Cat_74_mitochondrion_complete_genome

OR077823.1:10690-11307_Felis_catus_isolate_Cat_72_mitochondrion_complete_genome

OR077813.1:10690-11307_Felis_catus_isolate_Cat_58_mitochondrion_complete_genome

OR077810.1:10690-11307_Felis_catus_isolate_Cat_51_mitochondrion_complete_genome

OR077795.1:10690-11307_Felis_catus_isolate_Cat_36_mitochondrion_complete_genome

OR077789.1:10690-11307_Felis_catus_isolate_Cat_29_mitochondrion_complete_genome

OR077787.1:10690-11307_Felis_catus_isolate_Cat_27_mitochondrion_complete_genome

OR077785.1:10690-11307_Felis_catus_isolate_Cat_25_mitochondrion_complete_genome

OR077784.1:10690-11307_Felis_catus_isolate_Cat_23_mitochondrion_complete_genome

OR077783.1:10690-11307_Felis_catus_isolate_Cat_22_mitochondrion_complete_genome

OR077782.1:10690-11307_Felis_catus_isolate_Cat_21_mitochondrion_complete_genome]

[Hap_8: 4 OR085752.1:10691-11308_Felis_catus_isolate_Cat_57_mitochondrion_complete_genome OR077819.1:10691-11308_Felis_catus_isolate_Cat_66_mitochondrion_complete_genome

OR077803.1:10691-11308_Felis_catus_isolate_Cat_44_mitochondrion_complete_genome

OR077767.1:10691-11308_Felis_catus_isolate_Cat_105_mitochondrion_complete_genome]

[Hap_9: 1 OR077856.1:10690-11307_Felis_catus_isolate_Cat_P7_mitochondrion_complete_genome]

[Hap_10: 26 OR077855.1:10691-11308_Felis_catus_isolate_Cat_P6_mitochondrion_complete_genome OR077850.1:10690-11307_Felis_catus_isolate_Cat_P12_mitochondrion_complete_genome

OR077846.1:10691-11308_Felis_catus_isolate_Cat_BCB29_mitochondrion_complete_genome

OR077843.1:10690-11307_Felis_catus_isolate_Cat_99_mitochondrion_complete_genome

OR077840.1:10690-11307_Felis_catus_isolate_Cat_91_mitochondrion_complete_genome

OR077836.1:10690-11307_Felis_catus_isolate_Cat_87_mitochondrion_complete_genome

OR077826.1:10690-11307_Felis_catus_isolate_Cat_75_mitochondrion_complete_genome

OR077822.1:10690-11307_Felis_catus_isolate_Cat_71_mitochondrion_complete_genome

OR077821.1:10690-11307_Felis_catus_isolate_Cat_6_mitochondrion_complete_genome

OR077817.1:10690-11307_Felis_catus_isolate_Cat_63_mitochondrion_complete_genome

OR077816.1:10690-11307_Felis_catus_isolate_Cat_61_mitochondrion_complete_genome

OR077814.1:10690-11307_Felis_catus_isolate_Cat_5_mitochondrion_complete_genome

OR077811.1:10690-11307_Felis_catus_isolate_Cat_54_mitochondrion_complete_genome

OR077808.1:10690-11307_Felis_catus_isolate_Cat_4_mitochondrion_complete_genome

OR077805.1:10690-11307_Felis_catus_isolate_Cat_46_mitochondrion_complete_genome

OR077799.1:10690-11307_Felis_catus_isolate_Cat_3_mitochondrion_complete_genome

OR077794.1:10690-11307_Felis_catus_isolate_Cat_35_mitochondrion_complete_genome

OR077793.1:10690-11307_Felis_catus_isolate_Cat_32_mitochondrion_complete_genome

OR077792.1:10690-11307_Felis_catus_isolate_Cat_31_mitochondrion_complete_genome

OR077788.1:10690-11307_Felis_catus_isolate_Cat_28_mitochondrion_complete_genome

OR077777.1:10690-11307_Felis_catus_isolate_Cat_12_mitochondrion_complete_genome

OR077776.1:10690-11307_Felis_catus_isolate_Cat_11_mitochondrion_complete_genome

OR077773.1:10690-11307_Felis_catus_isolate_Cat_114_mitochondrion_complete_genome

OR077769.1:10690-11307_Felis_catus_isolate_Cat_107_mitochondrion_complete_genome

OR077766.1:10690-11307_Felis_catus_isolate_Cat_104_mitochondrion_complete_genome

**OQ096702.1:1-618_Felis_catus_isolate_Cat1_NADH_dehydrogenase_subunit_4_(ND4)_gene_partial_cds_mitochondrial**]

[Hap_11: 1 OR077838.1:10690-11307_Felis_catus_isolate_Cat_8_mitochondrion_complete_genome]

[Hap_12: 2 OR077781.1:10690-11307_Felis_catus_isolate_Cat_18_mitochondrion_complete_genome OR077774.1:10690-11307_Felis_catus_isolate_Cat_115_mitochondrion_complete_genome]

[Hap_13: 3 OR077841.1:10691-11308_Felis_catus_isolate_Cat_94_mitochondrion_complete_genome OR077833.1:10691-11308_Felis_catus_isolate_Cat_81_mitochondrion_complete_genome

MT499915.1:10989-11606_Felis_catus_voucher_C104-440-487_mitochondrion_complete_genome]

[Hap_14: 1 OR077824.1:10690-11307_Felis_catus_isolate_Cat_73_mitochondrion_complete_genome]

[Hap_15: 1 OR077779.1:10691-11308_Felis_catus_isolate_Cat_14_mitochondrion_complete_genome]

[Hap_16: 1 NC_001700.1:10985-11602_Felis_catus_mitochondrion_complete_genome]

1. MARGARIT (*Felis margarita*)

[Hap_17: 1 NC_028308.1:11002-11619_Felis_margarita_isolate_FMA_mitochondrion_complete_genome]

[Hap_18: 1 OR095102.1:10693-11310_Felis_margarita_mitochondrion_complete_genome]

1. NIGRIP (*Felis nigripes*)

[Hap_19: 1 NC_028309.1:10882-11496_Felis_nigripes_isolate_FNI_mitochondrion_complete_genome]

1. CHAUS (*Felis chaus*)

[Hap_20: 1 NC_028307.1:10789-11406_Felis_chaus_isolate_FCH_mitochondrion_complete_genome]

**Table S2:** Photo-trapped species of birds.

BIRDS species: Crane *(Grus grus)* (1); Pigeon *(Columba palumbus)* (2); Thrush *(Turdus philomenus)* (3); Blackbird *(Turdus merula)* (4); Buzzard *(Buteo buteo)* (5); Eurasian skylark *(Alauda arvensis)* (6); Partridge *(Alectoris rufa)* (7); Long-tailed Tern *(Cyanopica cyanus)* (8); Eurasian woodcock *(Scolopa rusticola)* (9); robin *(Erithacus rubecula)* (10); saithe *(Parus major)* (11); sparrow *(Paser domesticus)* (12); owl *(Athene noctua)* (13). The fences or sampling stations numbered 1 to 26 as in Table 1

| SAMPLING YEAR | DATE RANGE | TOTAL CAMERAS | SAMPLING FARM | TOTAL PHOTOS AND VIDEOS (MAMMALS AND BIRDS) | BIRDS SPECIES  OBSERVED |
| --- | --- | --- | --- | --- | --- |
| 2014 /2015 | December | 12 | El Tejarejo(1)  y Pizarro (2) y la Ventosilla (3) | 91,14/45,57 | *1(3),2(4), 3(1), 4(1)* |
|  | December | 8 | **El Canchal * (4)** | 87,25/43,625 | *5(1*) |
|  | January | 9 | La Perala (5) y Cabeza Gorda (6) | 172,66/86,33 | *3(1), 4(2), 5(1), 7(1), 8(1),* |
|  | January | 7 | Trinidade (7) y La Umbria (8) | 169,14/84,57 | *4(3), 3(1), 7(2), 3(1),* |
|  | February | 6 | Dehesa boyal de Santiago del Campo (9) | 158,33/79,1666 | *11(1), 2(1), 10(3), 4(3),* |
|  | March | 2 | El Edén (10) | 143/71,5 | *7(1), 4(1), 8(1),* |
|  | April | 8 | Parapuños (11) | 296,75/148,375 | *4(3), 10(1)*, 8*(2), 12(1), 7(1)* |
|  | April | 2 | El Cabril (12) | 50/54,5 |  |
|  | Mai | 9 | La Gama 13) | 638,88/319,4 (mucho viento) | *4(2)*, |
| 2015/2016 | December | 6 | **Valdeagudo *(14)** | 66/33 | *9(1)*, 2*(1), 4(1),* |
|  | December | 2 | **Las Mesas * (15)** | 44/22 |  |
|  | December | 6 | **La Mezquita * (16)** | 34/17 |  |
|  | December | 4 | **El Ahijón * (17)** | 58/29 | *4(1)* |
|  | January | 18 | **Moñigueros * (18)** | 74,22/37,11 | *4(1),* 2*(1)* |
|  | March | 17 | **Matavacas * (19)** | 79,05/39,52 | *4(4),* 2*(1), 8(2),* 7*(1)* |
| 2016/2017 | December | 13 | La Breña (20) | 51,23/25,61 | 4*(1),*  8*(1)* |
|  | January | 14 | Valdeposadilla (21) | 18,71/9,35 |  |
|  | February | 16 | Las Paredes (22) | 171,375/85,68 | *4(1)* |
| 2017/2018 | December | 15 | La Longuera (23) | 37,26/18,63 |  |
|  | January | 15 | El Castillo de Castellano de abajo (24) | 175,4/87,7 | 10*(1), 3(1)* |
|  | February | 15 | **El Castillo de Castellano de arriba * (25)** | 204/102 | *4(3),* 10*(1), 3(1),* 13*(1)* |
|  | April | 15 | Barrantes (26) | 60,8/30,4 | *4(3), 8(2), 3(5), 2(1)* |
