# Supplementary material for: The wildcat (Felis s. silvestris) in the Mediterranean forest: sighting through photo-trapping and non-invasive hair collection for genetic purposes
Source: Vet Res Commun. 2024 May 21;48(4):2309–20. doi: 10.1007/s11259-024-10402-3 (PMC11315778; doi:10.1007/s11259-024-10402-3)
Supplement: Supplementary file 2 — Supplementary file2 (DOCX 18 kb) [file 11259_2024_10402_MOESM2_ESM.docx]

Link:

Accession the video S3 via “Dehesa Repostorio Institucional. Universidad de Extremadura”.

<http://hdl.handle.net/10662/21208>
