# Supplementary material for: The wildcat (Felis s. silvestris) in the Mediterranean forest: sighting through photo-trapping and non-invasive hair collection for genetic purposes
Source: Vet Res Commun. 2024 May 21;48(4):2309–20. doi: 10.1007/s11259-024-10402-3 (PMC11315778; doi:10.1007/s11259-024-10402-3)
Supplement: Supplementary file 3 — Supplementary file3 (DOCX 271 kb) [file 11259_2024_10402_MOESM3_ESM.docx]

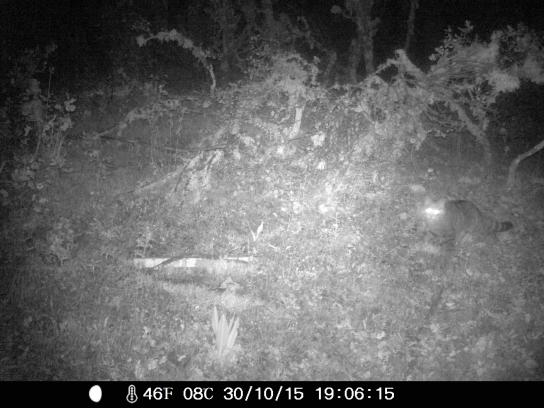


**Figure S2:** Wildcat *(Felis s. silvestris)* in the surroundings of the Garciaz river. Leaves no hair.


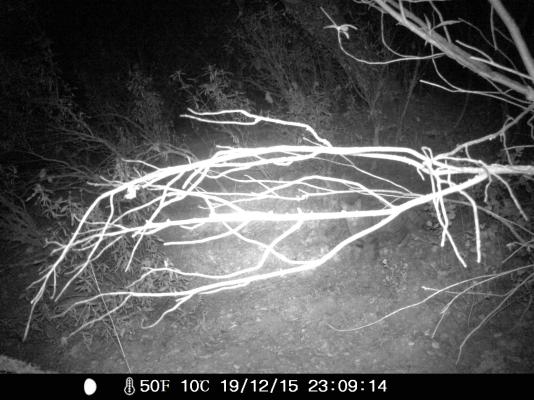


**Figure S3:** Wildcat *(Felis s. silvestris)* in the surroundings of the Garciaz river. Leaves no hair.


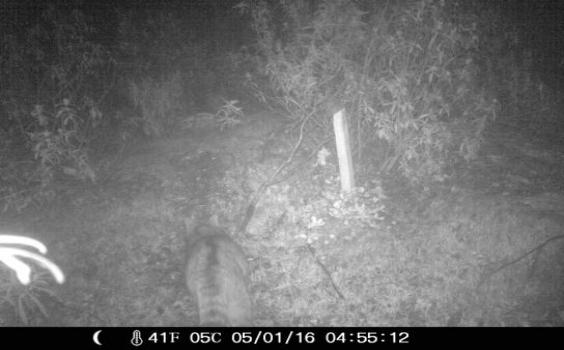


**Figure S4:** Wildcat *(Felis s. silvestris)* pregnant (possibly the same as figure S5 with two cubs in the hollow of an holm oak: figure S6) in the surroundings of the Garciaz river. Leaves no hair.


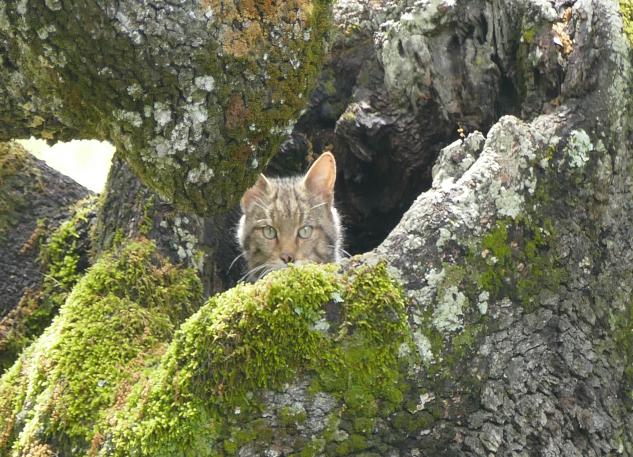


**Figure S5:** Wildcat *(Felis s. silvestris)* in its beak and possibly the same as figure S4.


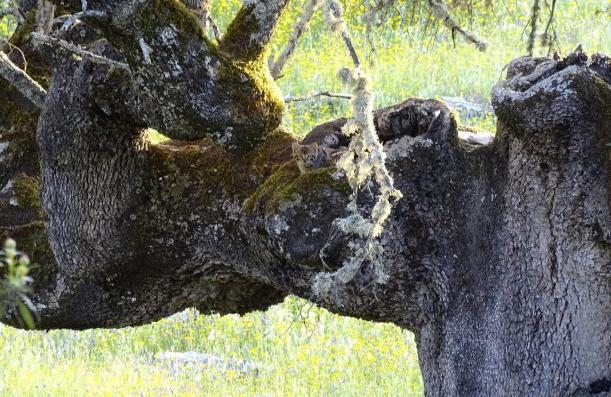


**Figure S6:** wildcat cub *(Felis s. silvestris*)
