# Supplementary material for: The wildcat (Felis s. silvestris) in the Mediterranean forest: sighting through photo-trapping and non-invasive hair collection for genetic purposes
Source: Vet Res Commun. 2024 May 21;48(4):2309–20. doi: 10.1007/s11259-024-10402-3 (PMC11315778; doi:10.1007/s11259-024-10402-3)
Supplement: Supplementary file 4 — Supplementary file4 (DOCX 344 kb) [file 11259_2024_10402_MOESM4_ESM.docx]

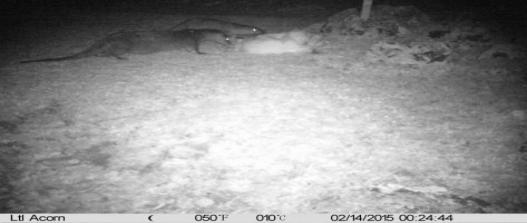


**Figure S7:** Otter (*Lutra lutra*)


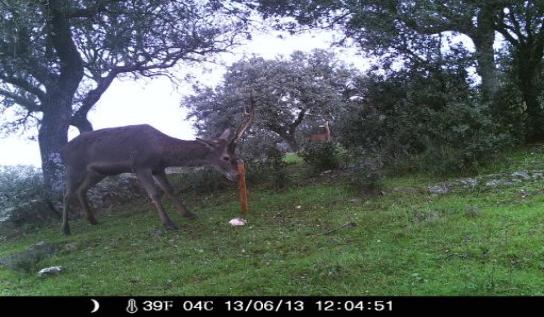


**Figure S8:** Red deer (*Cervus elaphus*).


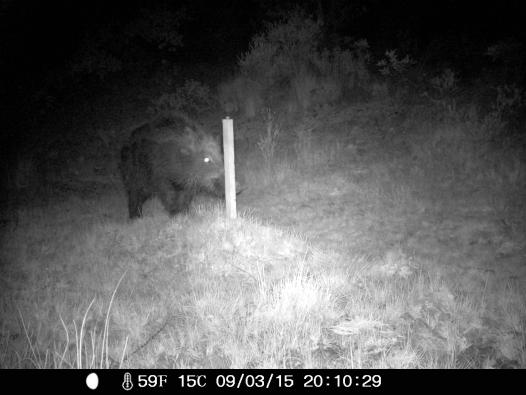


**Figura S9:**  Wild board (*Sus scrofa*).


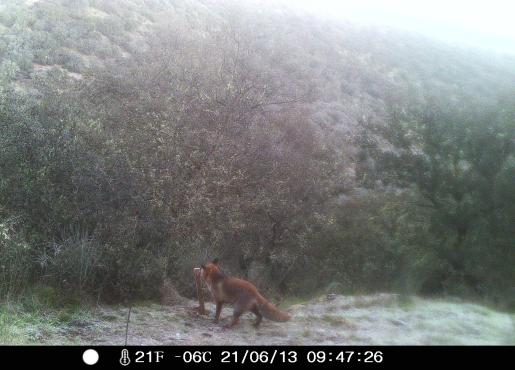


**Figure S10:** Fox (*Vulpes vulpes*).


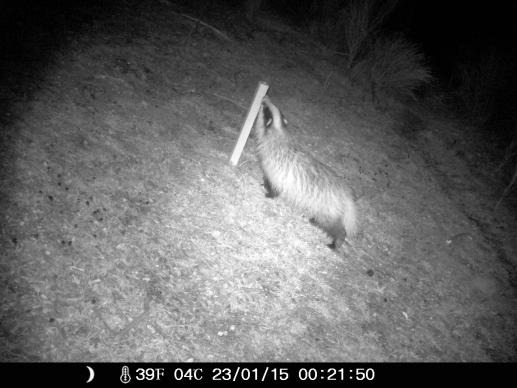


**Figure S11:** Badger (*Meles meles*).


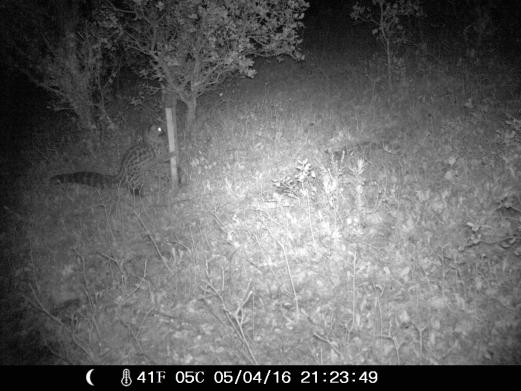


**Figure S12:** Genet (*Genetta genetta*).


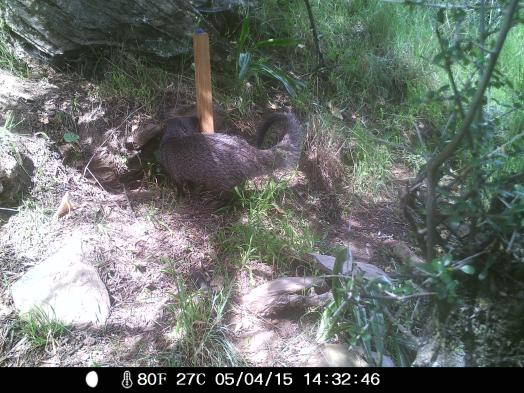


**Figure S13:** European mongoose *(Herpestes ichneumon*).


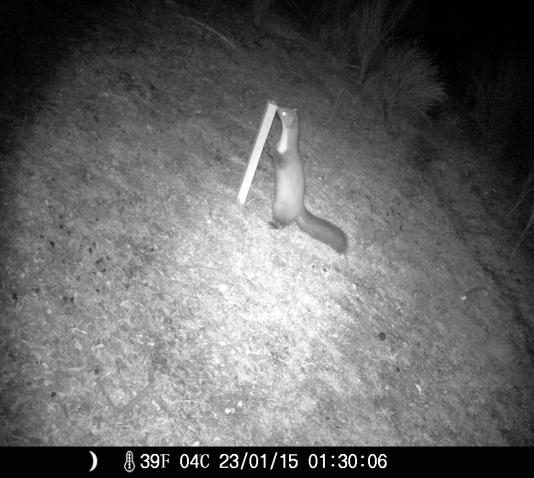


**Figure S14:** Beech marten (*Martes foina*).


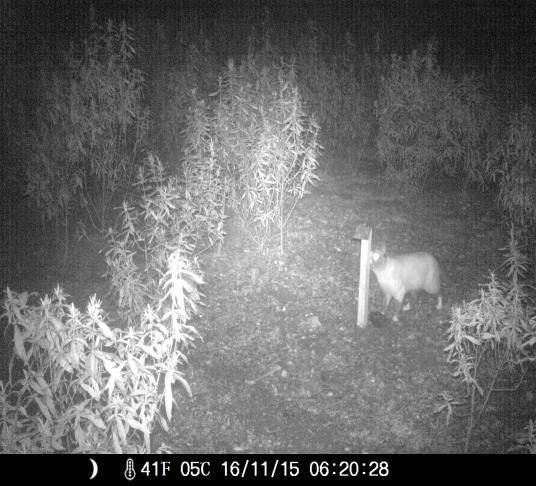


**Figure S15:** domestic cat (*Felis catus)*
